# In Situ Raman Characterization of SOFC Materials in Operational Conditions: A Doped Ceria Study

**DOI:** 10.3390/membranes10070148

**Published:** 2020-07-10

**Authors:** Cecilia Solís, María Balaguer, José M. Serra

**Affiliations:** 1 Instituto de Tecnología Química, Universitat Politècnica de València–Consejo Superior de Investigaciones Científicas, Avenida de los Naranjos s/n, 46022 Valencia, Spain; cecilia.solis@frm2.tum.de (C.S.); mabara@itq.upv.es (M.B.); 2 Heinz Maier-Leibnitz Zentrum (MLZ), TU München, Lichtenbergstr. 1, 85748 Garching, Germany

**Keywords:** Raman spectroscopy, doped ceria, in-situ Raman cell

## Abstract

The particular operational conditions of electrochemical cells make the simultaneous characterization of both structural and transport properties challenging. The rapidity and flexibility of the acquisition of Raman spectra places this technique as a good candidate to measure operating properties and changes. Raman spectroscopy has been applied to well-known lanthanide ceria materials and the structural dependence on the dopant has been extracted. The evolution of Pr-doped ceria with temperature has been recorded by means of a commercial cell showing a clear increment in oxygen vacancies concentration. To elucidate the changes undergone by the electrolyte or membrane material in cell operation, the detailed construction of a homemade Raman cell is reported. The cell can be electrified, sealed and different gases can be fed into the cell chambers, so that the material behavior in the reaction surface and species evolved can be tracked. The results show that the Raman technique is a feasible and rather simple experimental option for operating characterization of solid-state electrochemical cell materials, although the treatment of the extracted data is not straightforward.

## 1. Introduction

Raman spectroscopy is a quick and flexible method of characterizing structure and composition capable of providing direct, molecularly specific information regarding the physical and chemical processes occurring within functional solid oxide fuel cells (SOFCs) and electrolyzers (SOECs) in real time [1]. The main limitations of the technique are that the Raman scattering process is inherently weak (only a small fraction of photons is inelastically scattered, ca. 1 of 10^6^), and that not all species are Raman active on specific materials. Besides, the absence of a signal is not indicative of the absence of the specie and the fluorescence of a sample can hide the Raman signal of the target surface species.

The use of spectroscopic techniques for surface and solid-state studies is broadly reported since it allows the identification of the surface species present on the electrocatalyst surface, such as protons, hydrides, peroxides, and superoxides attached to different surface sites, i.e., atoms. Thus, it makes possible the determination of elemental mechanisms in the partial cathode and anode reactions of an electrolytic cell. The combination of surface spectroscopy, gas adsorption/exchange studies and catalysis and electrocatalysis tests may allow finding correlations between the surface species population and kinetics, and the final electrochemical performance of the SOFC and SOEC electrodes [2,3,4,5,6].

Raman spectroscopic studies of adsorbed species have been carried out with reasonable success on high area and relatively light adsorbents such as silica and alumina. However, to obtain spectra from species formed on oxide-supported metals has been proved to be much more difficult. This is particularly due to sensitivity, i.e., the metal particles have specific surface areas of the order of tens rather than hundreds of square meters per gram. Difficulties are also caused by the fact that metal preparations are either highly colored, absorbing most of the energy from the laser beam and being then locally raised in temperature (possibly by several hundred degrees), and then the adsorbate may be destroyed or desorbed [7].

In the materials typically used in SOFC surfaces, adsorbed species occur only under certain condition of temperature and atmospheric partial pressures, which indeed coincide with their operational conditions. In this paper, we have used the possibilities of the Raman techniques to ascertain two of the main aspects for the operability of the materials in a membrane for a fuel cell, i.e., the availability of oxygen vacancies and the in situ evaluation of the changes undergone by the material and evolved species in operation conditions. Lanthanide-doped ceria has been the system of choice for the Raman study due to the stability of the fluorite phase. Ceria is very tolerant to the dissolution of lanthanide cations, preserving the structure while modifying its properties [8]. Trivalent rare earths promote the ionic conductivity by introducing extrinsic oxygen vacancies in the lattice. The addition of elements with two valence states may increase the ionic conductivity, but also develop mixed ionic electronic conductivity, i.e., ceria doped with Tb and Pr. The lattice chemical expansion is produced by acceptor doping and/or reduced cation formation [9], which produces stresses or constrictions in the structure that affect the properties. These changes in the structure introduced by the dopant cation can be detected by Raman spectroscopy. Extra compositions by adding Co as a sintering aid are included to study its influence in crystal size and conductivity. For the second aim, the construction of a tailor made in situ Raman cell able to reproduce the operation conditions of an electrochemical cell will allow the quick and in situ evaluation of the surface evolution. The design and fabrication of such a Raman cell is reported. 

## 2. Materials and Methods

In order to synthesize powders of nanometric size, CeO_2−δ_, Ce_0.9_Ln_0.1_O_2−δ_ (Ln = La, Gd, Eu, Pr, Tb, Yb, Er) and 2%mol Co doped CeO_2−δ_ and Ce_0.9_Ln_0.1_O_2−δ_ were prepared by the coprecipitation method. Corresponding lanthanide nitrates were dissolved in distilled water at 50 °C and a solution of (NH_4_)_2_CO_3_ was added for precipitation. In the compositions where cobalt was required as sintering aid, it was added over the dried precursor powder by incipient wetness impregnation. This means that a cobalt nitrate solution in water was poured dropwise over the dry powders up to the pore volume. Finally, each powder was calcined for 5 h in air atmosphere at 800 °C to decompose the residual nitrates and carbonates and to form the fluorite phase [8,10]. 

For the in situ evaluation of materials under operational conditions, an oxygen permeation experiment was performed using a Ce_0.9_Gd_0.1_O_2−δ_ (CGO) dense membrane. This membrane was prepared by uniaxially pressing 26 mm diameter disc and subsequently calcinating at 1400 °C for 10 h to obtain a dense specimen. Afterwards both disk sides were screen printed with a 20 mm layer of a Pt ink (Mateck, Jülich, Germany), aiming to improve surface catalytic behavior.

Raman spectra, with a 2 cm^−1^ resolution, were collected with a Renishaw inVia Raman spectrometer (Malvern, UK) equipped with a Leica DMLM microscope and a 514.5 nm Ar^+^ ion laser and 785 nm Nd:YAG laser as excitation sources. A ×50 objective of 8 mm optical length was used to focus the depolarized laser beam on a spot of about 3 μm in diameter. The Raman scattering was collected with a charge coupled device (CCD) array detector.

Two different Raman cells were used in order to perform in situ experiments at high temperature in different atmospheres and by applying a current:

- A Linkan High-Temperature Catalyst Stage CCR1000 (Tadworth, UK). This stage allows heating the sample from room temperature (RT) up to 1000 °C, with heating rates from 1 to 130 °C/min and with a temperature stability of 1 °C. This stage also supports pressure up to 5 bar and has a quartz window installed.
- A new in situ Raman cell developed to measure in operando electrochemical cells, whose complete characteristics are explained in the next paragraphs and can operate from RT up to 600 °C with two independent gas chambers and electronic connectors.

To identify the crystalline phase and the lattice parameters of the samples, the powders were characterized by X-ray diffraction (XRD). The measurements were carried out on a PANalytical CubiX fast diffractometer (Malvern, UK), using CuKα1 radiation (λ = 1.5406 Å) and an X’Celerator detector in Bragg–Brentano geometry. XRD patterns were recorded in the 2θ range from 0° to 90° and analysed using X’Pert Highscore Plus software [11]. The lattice parameters were obtained by Rietveld refinement [12] of the diffraction patterns using the program *Fullprof* [13] suite Version 2017 by applying a Thompson–Cox–Hastings pseudo-Voigt function. A LaB_6_ sample was measured in order to obtain the instrument resolution file and to get the apparent size by applying the Scherrer formula [14] considering both Gaussian and Lorentzian components [13].

Total conductivity measurements were performed on rectangular probes (4 × 0.4 × 0.2 cm^3^) of the fluorite powders, which were uniaxially pressed 1 min at 125 MPa and subsequently sintered for 5 h at 1300 °C in air. Electrical conductivity measurements were conducted with a standard four-point DC technique on the sintered rectangular bars using silver wire and paste for electrical contact. The measurements were carried out in the temperature range from 400 to 800 °C by cooling (1 °C/min) in air. The constant current was supplied by a programmable current source (Keithley 2601, Cleveland, OH, USA) and the voltage drop across the sample was measured by a multimeter (Keithley 3706). Prior to measurements, the samples were equilibrated for 2 h at 800 °C.

## 3. Results and Discussion

### 3.1. CeO_2−δ_, Ce_0.9_Ln_0.1_O_2−δ_ (Ln = La, Pr, Eu, Gd, Tb, Yb) and 2%mol Co Doped CeO_2−δ_ and Ce_0.9_Ln_0.1_O_2−δ_ Nanoparticles

**Figure 1:**
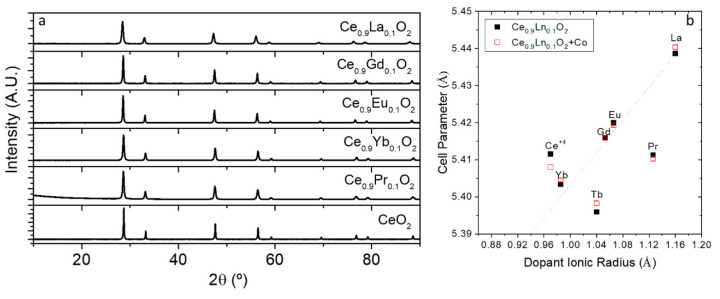
(**a**) X-ray diffraction (XRD) patterns of Ce_0.9_Ln_0.1_O_2−δ_ powders with Ln = Pr, Yb, Eu, Tb, Gd and La as prepared at 800 °C and (**b**) cell parameter as a function of the 3+ the dopant ionic radii (except for Ce, in which the value of Ce^4+^ is represented).

Figure 1a shows the XRD patterns of the as synthesized Ce_0.9_Ln_0.1_O_2−δ_ materials at 800 °C. They all correspond to a cubic Fm-3m symmetry without any traces of impurities or secondary phases. The cell parameters were obtained by Rietveld refinement and are summarized in Table 1 together with those of the 2%mol Co doped (all Rietveld refined patterns can be found in Supporting Figure S1). 

The Ce_0.9_Ln_0.1_O_2_**_-δ_** lattice parameter directly correlates with the ionic size of the dopants (also fully detailed in Table 1, taken in 3+ oxidation state for 8-fold coordination except for Ce where the 4+ oxidation state was selected [15]. This can be graphically seen in Figure 1b where the cell parameter of each ceria is plotted as a function of the aforementioned ionic size of the dopants in the 3+ oxidation state (except for Ce). From the graph it is easily seen that Pr and Tb should be partially in 4+ oxidation state (ionic radii of 0.96 Å and 0.88 Å for Pr^4+^ and Tb^4+^, respectively) in order to justify the cell parameter obtained. Considering this, it is possible to estimate the amount of the tetravalent cations (by Vegard’s slope), which gives rise to 60% and 38% of tetravalent cations for Pr and Tb, respectively [8].

**Figure 2:**
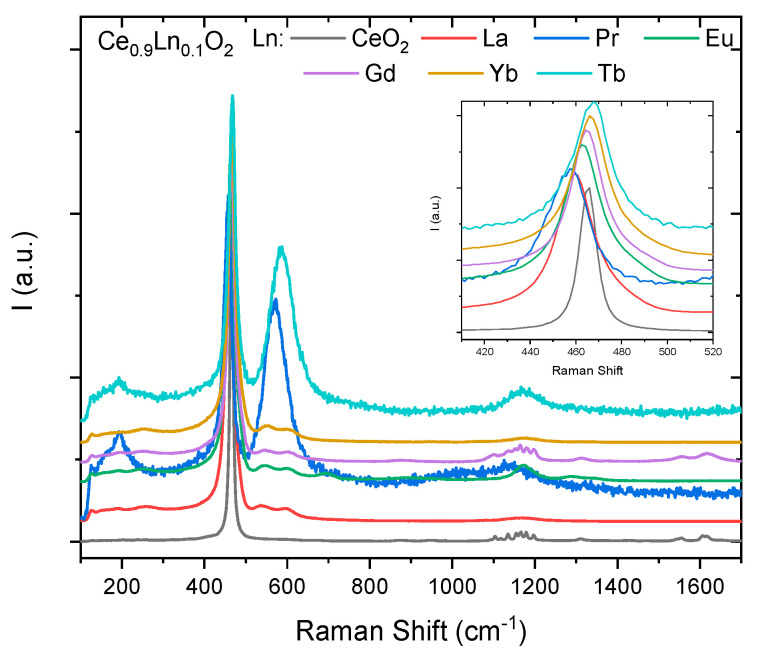
Raman spectra of the Ce_0.9_Ln_0.1_O_2_ (Ln = (Ln = La, Pr, Eu, Gd, Tb, Yb)) doped samples (sintered at 800 °C) from 100–1700 cm^−1^ and magnification around ~464 cm^−1^.

This correlation is also followed by Raman spectroscopy. Figure 2 shows the Raman spectra of the Ce_0.9_Ln_0.1_O_2**-δ**_ (Ln = La, Pr, Eu, Gd, Tb, Yb) and 2%mol Co doped Ce_0.9_Ln_0.1_O_2**-δ**_ samples from 100–1700 cm^−1^. It is also represented a zoom of the main fluorite Raman peak (F_2g_ mode) at ~464 cm^−1^. As said before, cerium oxide has a cubic fluorite structure, and belongs to the $O_{h}^{5}$ (F*m-3m*) space group. This structure has six optical-phonon branches, which yield three zone-center frequencies. These frequencies correspond to the doubly degenerated transverse optical (TO) mode (272 cm^−1^), the triply degenerated Raman-active mode (465 cm^−1^), and the non-degenerated longitudinal optical (LO) mode (595 cm^−1^) [16,17]. The single allowed Raman mode has F*_2g_* symmetry and can be understood as a symmetric breathing mode of the O atoms around each cation (Ce-O_8_ unit). For undoped ceria, this mode appears at approximately 465 cm^−1^. Therefore, this unit should be very sensitive to a disorder in the oxygen sublattice resulting from thermal and/or grain size-induced non-stoichiometry.

Ln-doped CeO_2−__δ_ compounds (Ln_x_Ce_1−x_O_2−δ_) have shown a small systematic shift of the F_2g_ mode frequency regarding pure CeO_2−δ_, as can be seen in the zoom shown in Figure 2. In addition, for all the doped samples the F_2g_ peak becomes wider. The lattice disorder induced by the substitution of Ln^3+^ ions causes this shape change in the Raman line, which can be studied using the spatial correlation model. The spatial correlation model is a model that quantitatively accounts for the details of the first-order Raman line shape (broadening and broadening asymmetry) and that has to be taken into account in nanocrystalline systems due to the changes in their vibrational properties related to the grain size spatially confined effects. It analyzes the Raman line shape and obtains lattice disorder information [18,19].

An additional peak shoulder at ~570 cm^−1^, close to the F_2g_ mode, has been attributed to the formation of oxygen vacancies. The higher relative intensity observed for Pr and Tb of the ~570 cm^−1^ shoulder is ascribed to the higher optical adsorption for these colored compounds [20]. The vibrational mode at around 600 cm^−1^ is attributed to oxygen vacancies’ defects (both intrinsic and extrinsic) induced by doping with Ln^3+^ [21,22]. Therefore, the band at 600 cm^−1^ is a consequence of either an ordering effect in the oxygen vacancies to produce confinement of the phonons within an inhomogeneous distribution of domains or structural distortions of the O sublattice. 

**Figure 3:**
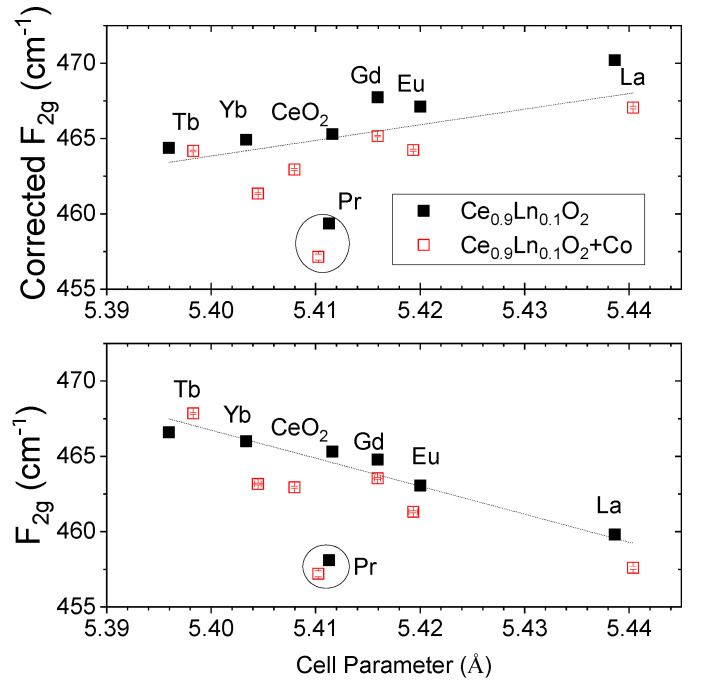
F_2g_ Raman shift as a function of the as measured cell parameter (**bottom**) and calculated F_2g_ Raman shift after Grüneisen parameter correction (**top**).

Figure 3 shows the shift of the F_2g_ mode as a function of the lattice parameter. We can find in the literature at least three different mechanisms contributing to the frequency shifts of the F_2g_ mode:

1. Dilation or contraction of the lattice [23]: the frequency shift Δω produced by a change in the lattice parameter Δa can be written in terms of the Grüneisen parameter:
  (1) $$\Delta \omega =-3\gamma \omega_{0}\Delta a/a_{0}$$
  where ω_0_ and *a*_0_ are the Raman frequency and the lattice parameter for CeO_2−δ_ respectively, and γ≡(B/ω)dω/dP is the Grüneisen constant (1.24 for CeO_2__−δ_ [20])
2. Particle size effects when it is <100 nm: the F_2g_ mode shifts to progressively lower energies and the line shape gets broader and asymmetric (on the low energy side) as the particle size gets smaller [24]. The increasing lattice constant with decreasing particle size (due to increased concentrations of point defects with decreasing particle size [25] explains satisfactorily this shift. The line width change can be explained by the inhomogeneous strain broadening associated with dispersion in particle size and by phonon confinement. The increase in its asymmetry is attributed to reduction of the phonon lifetime in the nanocrystalline regime [17].
3. Increase of the O vacancies: the increasing disorder due to oxygen vacancies shifts up in frequency the F_2g_ mode [20,23].

All three effects that can take part in these materials due to the different ionic radii of the introduced lanthanide and the nanometer size of the particles will be discussed. 

The effect of the cell parameter change can be seen in Figure 3, where the as measured Raman shift is plotted against the cell parameter (bottom) and also the shift after taking into account the Grüneisen parameter correction (top). The dependency of the F_2g_ with the lattice parameter changes from a negative slope of –186 (±30) as measured to a positive slope of 100 (±30) after taking into account the Grüneisen parameter correction. The variation on the F_2g_, even taking into account the Grüneisen parameter, indicates that more factors than the dilation or contraction of the lattice need to be considered to explain the obtained Raman shifts. It should be pointed out that Pr-doped ceria, however, does not obey the same trend due to the high amount of tetravalent cations for Pr (60%), as already shown in Figure 1b. The mixed oxidation state (3+/4+) characteristic of this dopant at high oxygen partial pressure (as in air)makes possible to register the reduction of Pr with increasing temperature and the changes in oxygen sublattice non-stoichiometry by following the oxygen vacancy related Raman peak mode, which will be developed in Section 3.2.

**Figure 4:**
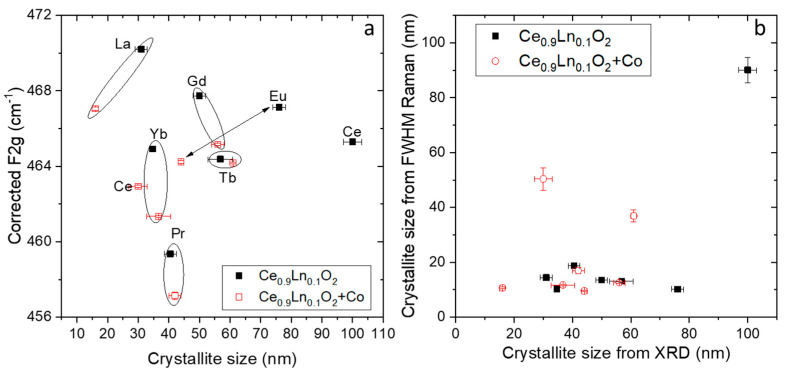
F_2g_ Raman shift as a function of the crystallite size (**a**) and obtained crystallite size from FWHM of the F_2g_ mode by applying equation (**b**) as a function of the X-ray diffraction (XRD)-obtained crystallite size (Table 1) for all the Ce_0.9_Ln_0.1_O_2__−δ_ and Co doped Ce_0.9_Ln_0.1_O_2__−δ_ samples.

Regarding the particle size effect, Figure 4a shows the F_2g_ Raman shift after Grüneisen parameter correction as a function of the crystallite size obtained from XRD by applying the Scherrer formula. Although a shift to decreasing energies when reducing crystallite size is expected, this effect is not clearly found in our materials, therefore the observed changed in the analyzed Raman shift will need to be related only to the presence of oxygen vacancies. Moreover, it has been also reported that the line width of the F_2g_ mode depends on the particle size, and for nanocrystalline CeO_2−δ_ the relationship has been empirically found for different authors as [19,26]:

Γ(cm^−1^) = 10 + 124/d (nm) (2)

where Γ is the FWHM (full width at half maximum) and d the crystallite size, measured by XRD (one example of a scanning electron microscope (SEM) image can be also found in Supporting Figure S2 for comparison). 

Figure 4b represents the obtained crystallite size from the FWHM of the F_2g_ mode by applying (2) as a function of the crystallite size obtained from X ray analysis by applying Scherrer formula (Table 1) for all the Ce_1−x_Ln_x_O_2−δ_ (black filled squares) and Co doped Ce_1−x_Ln_x_O_2−δ_ (red empty squares). As shown in the graph, the expected sintering effect of cobalt has not been observed at these low synthesis temperatures and the crystal sizes seem to be independent of the Co addition. Raman line widths were obtained by a fit of each spectrum to a Lorentzian line shape. It can be observed that only for CeO_2−__δ_ and Co-doped CeO_2−__δ_ are the obtained crystallite sizes similar regardless the method, while for all the Ln doped materials the sizes from Raman line width are smaller than expected. This is due to broader Raman peaks that expected for compounds with only particle size contributions, which means that the oxygen vacancies should contribute to the Raman peak width in all the Ln-doped materials (which is not observed in pure CeO_2−__δ_).

Finally, regarding the third effect related to the oxygen vacancies, it is well known that the introduction of Ln^3+^ ions causes the F_2g_ peak to become asymmetric and the appearance of a weak shoulder on the high frequency side that evolves into the observed broad peak at ~570 cm^−1^ (see Figure 2). This weak shoulder is associated with defect species with Oh symmetry, more specifically the existence of ${\mathrm{Ce}}^{3+}-V_{O}^{**}$ complexes in the ceria lattice [20,22,27]. In contrast, in the Pr and Tb doped samples the F_2g_ lines remains symmetric and although there is also a broad peak near 570 cm^−1^, this is relatively higher in intensity and narrower than the corresponding in the other Ln doped samples. In this case, as mentioned before, it is due to the higher optical adsorption for these colored compounds and the role of the Ln4+ oxidation state not present in the other Ln [20]. 

**Figure 5:**
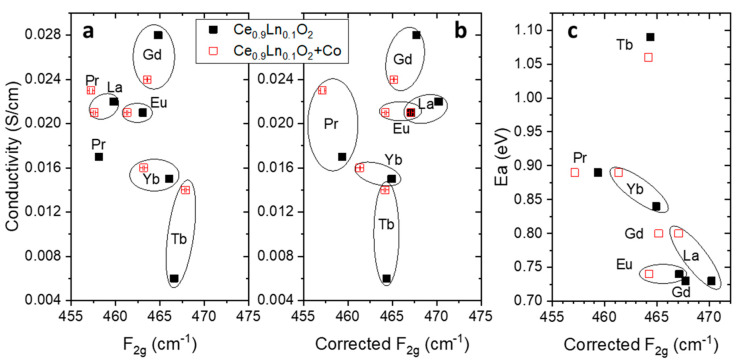
(**a**) Conductivity at 700 °C in air as a function of the F_2g_ Raman position and (**b**) after Grüneisen parameter correction Raman shift. (**c**) Activation energy dependence on corrected F_2g_ for Ce_0.9_Ln_0.1_O_2__−δ_ and Co-doped Ce_0.9_Ln_0.1_O_2__−δ_.

The presence of the oxygen vacancies can be unambiguously discerned from the high-temperature transport properties of the studied materials. Since doped cerias are mainly ionic conductors, it is possible to relate the lattice structural ordering modifications and oxygen vacancies concentration with the electrochemical properties [8,28,29,30]. Figure 5a shows the total conductivity of the different samples as a function of the as measured F_2g_ mode position, where the conductivity just decreases as the Raman shift increases. This tendency means that the conductivity is influenced by the size of the dopant cation, with a maximum observed for Gd^3+^. This maximum has been related to the less distorted cell observed for Gd regarding the rest of the lanthanides [8], and it is confirmed by the smallest F_2g_ shift from CeO_2−δ_ (see also Figure 3). Concerning the addition of Co, the influence in the conductivity depends on the main transport carriers. Sintering aids as CoO_x_ in cerias increase the crystal size of the material when sintered at high temperatures (it was demonstrated before that this is not happening when calcining at 800 °C) and segregate to the grain boundaries [8,29]. Trivalent lanthanide-doped cerias are ionic conductors, so the decrease in conductivity is due to the blocking of the oxide ions between grains. Differently, since the multivalent oxidation state of Pr and Tb turns these doped cerias mixed ionic and electronic conductors, Ce_0.9_Pr_0.1_O_2−δ_ and Ce_0.9_Tb_0.1_O_2−δ_ are benefited by the CoO_x_, which boost the electronic transport through the grain boundaries and enhance the total conductivity [8].

As mentioned in the previous paragraphs, observed changes on Raman shifts in these samples are produced not only by changes in the lattice parameters but also in the oxygen vacancies (no effects due to the crystallite size were demonstrated). When the influence of the lattice parameter is removed, we obtain the corrected F_2g_ Raman shift, shown in Figure 5b. Here, we can still distinguish a general trend with the Raman shift, except for Pr- and Tb-doped compounds. The mixed valence of Pr (calculated to be 60% as Pr4+ at room temperature), gives rise to mixed ionic-electronic conductivity and then to a higher total conductivity than that purely associated to its oxygen vacancy concentration (which in this case is related to the corrected F_2g_ Raman shift) [29]. The effect of the mixed conductivity can be also observed in the case of Tb, in which the Co addition has been demonstrated to produce an increase of the electronic conductivity while keeping the same ionic transport [28]. This is proved by the fact of getting a similar corrected F_2g_ Raman shift but higher total conductivity in Co-added Ce_0.9_Tb_0.1_O_2−δ_ than in bare Ce_0.9_Tb_0.1_O_2__−__δ_.

In order to properly discuss the conductivity dependence on corrected F_2g_, the Figure 5c plots the corresponding activation energies (Ea) of each sample as a function of the corrected F_2g_ Raman shift. Highest conductivity corresponds to lowest Ea value, obtained for Gd3+ and the trivalent dopants with longer cell parameters also show the higher conductivities, attributed to the decrease of Coulombic interactions. Since the size effect of the ionic radii is removed in the corrected Raman shift, the Ea dependency is attributed to the different association energy (Eass) caused by the ionic radius of the oxygen vacancies distortion [8,23].

### 3.2. High-Temperature Raman

**Figure 6:**
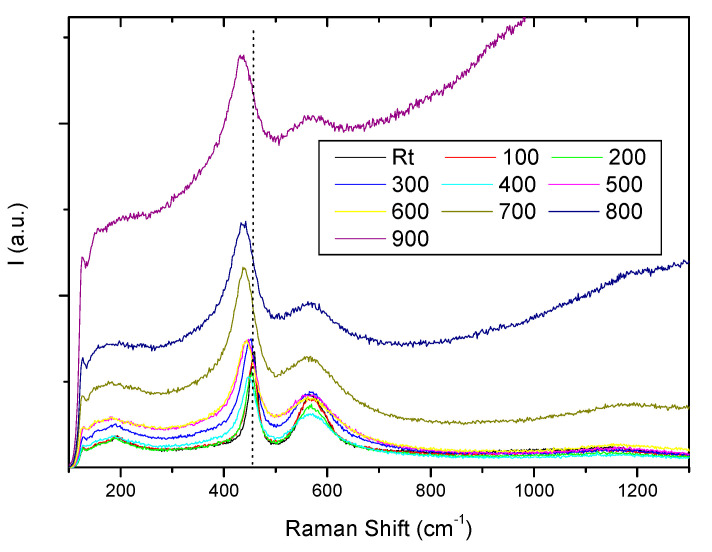
Raman spectra recorded at different temperatures in air, from RT up to 900 °C.

To properly correlate the Raman spectra with the high-temperature transport properties of the doped ceria we have selected Ce_0.9_Pr_0.1_O_2−δ_ to study Raman changes with temperature. For the purpose, a Linkan High-Temperature Catalyst Stage CCR1000 that allows heating the sample was employed. A heating rate of 5 °C/min was followed up to 900 °C, with a temperature stability of 1 °C. Figure 6 shows all Raman spectra recorded at different temperatures in air, from RT up to 900 °C. The vertical dot line in the graph indicates the Raman peak position at RT. As temperature increases, Pr oxidation state reduces as oxygen is released and oxygen vacancies are formed. By increasing the concentration of oxygen vacancies and therefore the non-stoichiometry in the lattice, the F_2g_ peak becomes asymmetric and the shoulder at ~570 cm^−1^, associated with defect species, broadens and shades at high temperature as the lattice becomes more mobile. Although the Raman peak width changes with the temperature, a peak shift is ascertained.

There are two fundamental contributions to the shift of the Raman peaks with temperature [31]:

1. Contribution due to the changes in vibrational amplitude of the atoms (change in occupation of the phonon states), “explicit” shift.
2. Contribution due to the change of the interatomic distances when the temperature changes, “implicit” or “volumetric” shift.

As the temperature increases, cell volume increases [29] due to the thermal expansion (as can be seen in Supporting Figure S3) and the Raman bands shift as a consequence of the piezospectroscopic effect. These two contributions can be expressed as:

(3) $${\left(\frac{\delta v}{\delta T}\right)}_{P}={\left(\frac{\delta v}{\delta T}\right)}_{V}+{\left(\frac{\delta v}{\delta V}\right)}_{T}{\left(\frac{\delta v}{\delta T}\right)}_{P}={\left(\frac{\delta v}{\delta T}\right)}_{V}-\frac{\alpha }{\beta }{\left(\frac{\delta v}{\delta P}\right)}_{T}$$

where *ν* is the Raman frequency, *T* is the temperature, *P* is the pressure, *V* is the volume and ${\left(\frac{\delta v}{\delta T}\right)}_{V}$ and ${\left(\frac{\delta v}{\delta V}\right)}_{T}{\left(\frac{\delta v}{\delta T}\right)}_{P}$ are the explicit and volumetric contributions respectively; $\alpha =\left(\frac{1}{V}\right){\left(\frac{\delta V}{\delta T}\right)}_{P}$ volume expansivity and $\beta =\left(\frac{1}{V}\right){\left(\frac{\delta V}{\delta P}\right)}_{T}$ compressibility.

In addition to these fundamental mechanisms, there is a contribution due to thermal expansion mismatch strain when the sample is constrained, for instance on a substrate as in thin films (although it was not considered in this case). 

**Figure 7:**
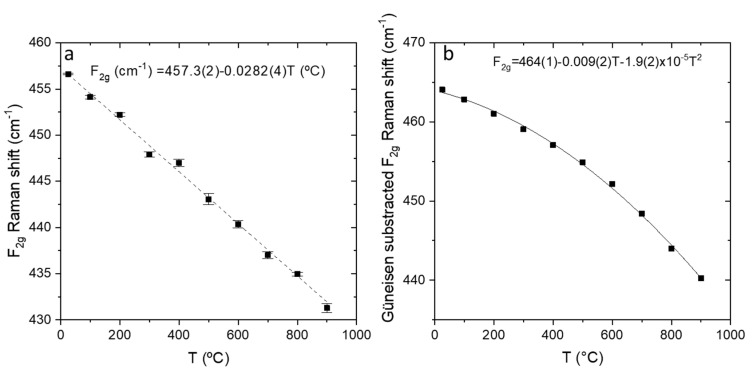
(**a**) F_2g_ Raman shift measured and (**b**) after subtracting Grüneisen shifts as a function of temperature.

Volume changes are due to the thermal expansion. Changes in Raman shift as measured can be seen in Figure 7a and after volume corrections by calculating the Grüneisen shifts in Figure 7b. It can be observed that after considering the volume cell contribution the effects are not linear, there are anharmonic effects.

**Figure 8:**
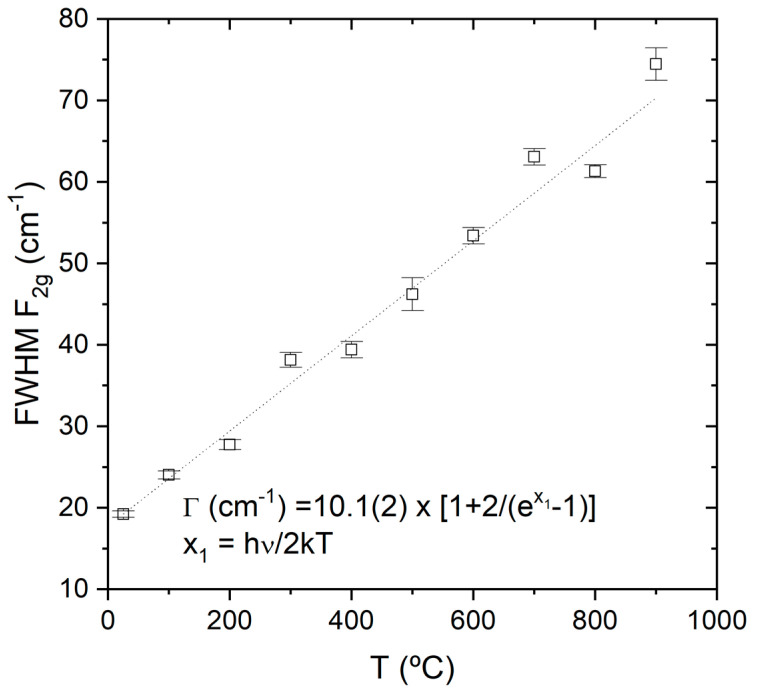
Full width at half maximum (FWHM) of the F_2g_ mode as a function of the temperature.

Raman bands broaden with increasing temperature as a result of the reduced phonon lifetime. This broadening has been modelled by Hart et al. [32] and successfully applied to silicon and yttria-stabilized zirconia [33]. In this model it is assumed that the lifetime of the optical phonon is limited by its decay into two acoustic phonons, with energy of one half of that of the optical phonon. According to this model, the temperature dependence of the half-width, Γ can be expressed as:

(4) $$\Gamma \left(T\right)=\Gamma_{0}\left[1+\frac{2}{\exp\left(h\nu_{0}/2kT\right)-1}\right]$$

where Γ_0_ is the linewidth at T = 0 K, *h* is Plank constant, *k* Boltzmann constant and ν_0_ the center band position of the mode. As can be observed in Figure 8, this equation describes perfectly the observed dependence of the line width. The curve displayed in Equation (4) corresponds to the best fit based on Equation (4), where only the Γ_0_ parameter was left free to vary, and it was obtained a Γ = 10.1(2) cm^−1^ that corresponds to a lifetime τ = 1.05 ps. 

**Figure 9:**
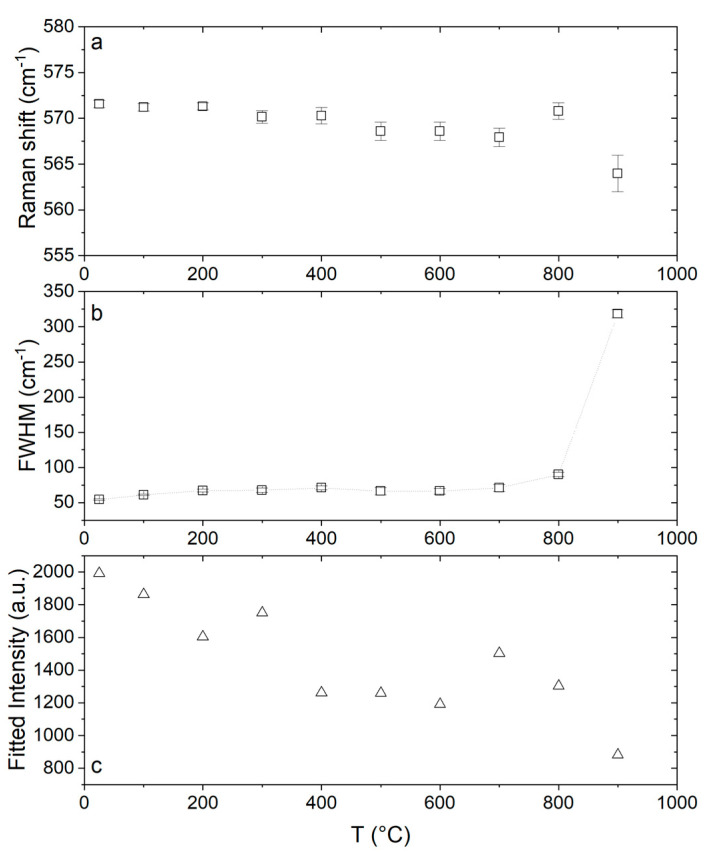
(**a**) Vacancies peak shift, (**b**) width and (**c**) intensity as a function of temperature.

When focusing on the effect of the temperature on the defect associated vibrational mode at ~570 cm^−1^, we can see the influence of the temperature on the Raman shift, the FWHM and the intensity in Figure 9a–c, respectively. While the changes on Raman shift and FWHM can be related to the aforementioned changes in the cell volume and lifetime of the phonons, the decrease in intensity with the temperature can be assigned to dissociation of oxygen vacancies from $M_{Ce}^{\prime }-V_{O}^{**}$ defect cluster. The isolation of defects leads to a decrease in intensity [22].

### 3.3. In Situ Raman Characterization of Solid Oxide Fuel Cell (SOFC) Materials at Operational Conditions: A Doped Ceria Study

**Figure 10:**
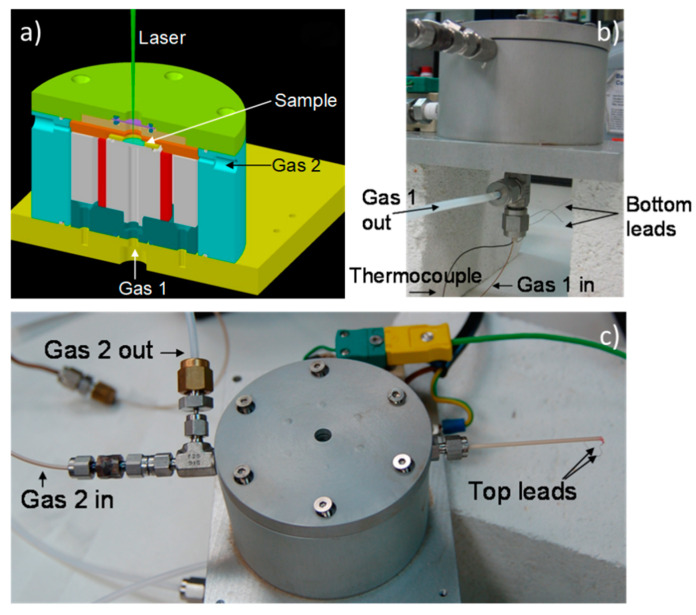
(**a**) CAD drawing, (**b**) front and (**c**) top view of the new fuel cell Raman stage.

One of the most interesting features of Raman spectroscopy is the rapidness to obtain a full measurement and, therefore, the possibility of registering structural changes as the material is operating in an electrolytic cell. To be able of such measurements, we built a home-designed Raman cell. The main characteristic of this new Raman cell is to perform Raman spectroscopy at high temperature during a complete electrochemical process of a cell, which implies two different atmospheres (anode and cathode) and electronic connections. This first version of a cell will measure different SOFC, SOEC, mixed conducting membranes, and materials under close-to-operation conditions (up to 600 °C). The cell consists of a two-chamber stage that are gastight insulated by the electrolyte. In Figure 10, the CAD drawing illustrates the assembly of the different elements in the stage (a) and two pictures of the different views of the new SOFC Raman stage, one from the front (b) and the other one from the top (c). The disc-shaped electrolyte placed in a holder separates two independent atmospheres. The upper gas inlet, the sweep or fuel, occurs throw an alumina tube, parallel to the upper side of the membrane or cathode. The lower side, the feed, is perpendicular to the lower side of the membrane through a silica capillary, while the rejection vents through a polytetrafluoroethylene (PTFE) tube.

**Figure 11:**
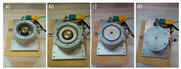
Details of the fuel cell Raman stage for in situ measurements: holder (**a**), insulation parts (**b**,**c**), and quartz window (**d**).

More details of the inside of the stage can be observed stepwise in Figure 11: the sample holder, the heating element and the gas 2 inlet, ceramic insulator elements, and the quartz window. The design permits to introduce two different gases in the stage (from the bottom and from the top, also visible in Figure 10b,c, respectively), heat the sample up to 600 °C and do different conductivity measurements with the 2 wires connected to the top electrode and other 2 wires to the bottom one. 

**Figure 12:**
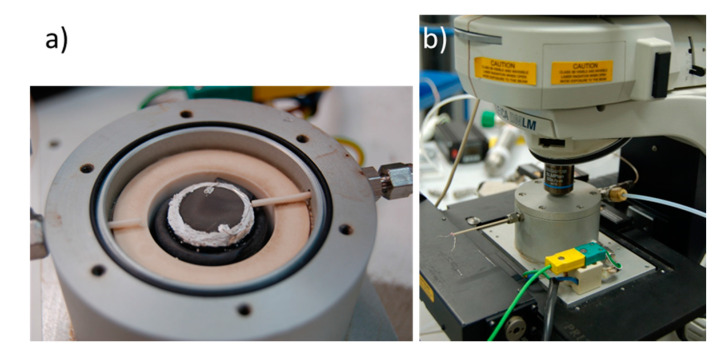
Ni/YSZ sample and electric connections in the new Raman stage (**a**) and the new SOFC Raman stage under the microscope (**b**).

The electrolyte or membrane sample placed on the holder is sealed using a Al_2_O_3_-based ceramic cement, thus becoming the separator between two independent chambers to flow different gases. Figure 12a represents a picture of a Ni/YSZ (yttria-stabilized zirconia) sample sealed on the holder. In the picture, the details of the electric connections, the alumina tubes used for the gas inlet, and the Ag leads can be observed. Finally, the cell is sealed by the aluminum tip where the quartz window is placed and Figure 12b shows a picture of the SOFC Raman stage under the microscope, ready for Raman scanning.

**Figure 13:**
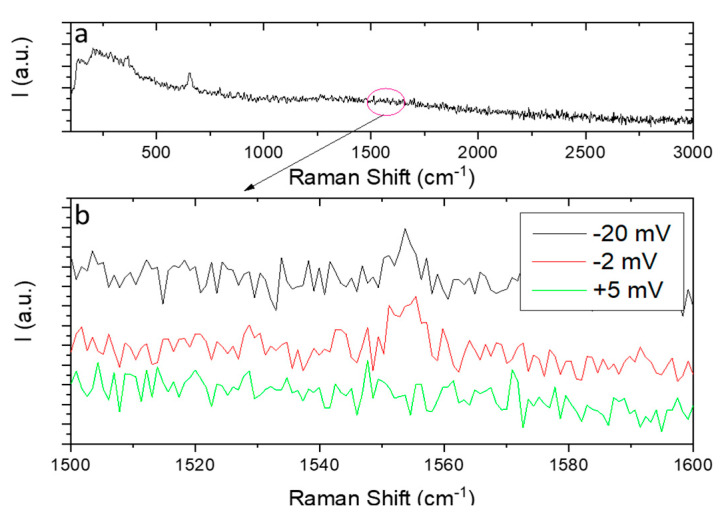
Raman spectra at 200 °C of a Pt/CGO/Pt symmetrical cell full range (**a**) and a zoom in from 1500–1600 cm^−1^ under different voltages (**b**).

This developed Raman stage was used for in situ monitoring oxygen species. Other authors have shown the influence of gadolinium doping on the structure and defects of ceria under fuel-cell operating temperature [30]. Figure 13 shows the Raman spectra from the top of a Pt layer in reducing atmospheres at 200 °C using a Ce_0.9_Gd_0.1_O_2__−__δ_ (CGO) membrane in the new home-made Raman cell developed in this work. The Pt/CGO/Pt cell was measured with reducing atmosphere in one side (Ar) and air in the other side and was analyzed under different applied voltages in order to pump oxygen from the oxygen-rich side to the other. 

The full Raman spectra of the symmetrical cell at 200 °C can be seen in Figure 13a and a zoom in the range where the oxygen vibrations modes were expected in Figure 13b, where the spectra under different applied voltages are compared. The Pt layer does not allow us to measure the Raman peaks associated to the CGO, and the only observed ones are due to the experimental set up (Al_2_O_3_). When applying –2 mV and –20 mV voltages we can see the O_2_ vibration mode at around 1555 cm^−1^ [34,35,36], which cannot be seen when applying the voltages in the other direction (+ 5 mV). Although some authors have related a peak around 1580–1597 cm^−1^ to graphitic carbon formation on the surface [37,38,39], the absence of other carbonate peaks rules out this possibility. Therefore, this demonstrates in situ the pumping of oxygen through the CGO from the oxygen atmosphere chamber to that of the reducing atmosphere, in which the measurement was performed. When increasing the temperature to close to operational conditions, although the oxygen pumping is improved, the obtained peaks become broader and more difficult to analyze. This technique within the use of this electrochemical home-made Raman cell constitutes a promising technique for in situ observation of this and similar species produced under electrochemical measurements, although the amount of transported species, in this case the amount of oxygen, cannot be quantified.

## 4. Conclusions

The versatility of Raman spectroscopy in the study of materials for fuel-cell applications under close to operational conditions has been reported. The very diverse structural information that can be extracted from a Raman spectrum includes the modifications in the crystal parameters, the influence of particle sizes, and the formation of oxygen vacancies.

By a systematic doping of ceria with different lanthanides, it has been possible to correlate the Raman shift with the structural changes of the cubic fluorite cell produced by the dopant. Furthermore, the study of the Pr doped ceria at high temperature has shown the strengthens of the Raman technique in order to follow in situ changes with the temperature (cell volume change due to purely thermal effects and also effects due to oxygen vacancies’ formation) that afterwards can be related to cell expansion and electrochemical properties.

Finally, a new home-made Raman cell designed to perform in situ measurements under operational conditions (high temperature, two gas chambers and electrical connections) has been shown, opening the possibility of in situ measurement of surface species formed during the electrochemical performance of different materials.

## Figures and Tables

**Table 1:** Cell parameter and crystallite size of the different analyzed materials obtained by Rietveld refinement and ionic radius of the dopant in 3+ oxidation state (* except for Ce which is considered the 4+).

	CeO_2−δ_	Ce_0.9_La_0.1_O_2−δ_	Ce_0.9_Pr_0.1_O_2−δ_	Ce_0.9_Eu_0.1_O_2−δ_	Ce_0.9_Gd_0.1_O_2−δ_	Ce_0.9_Tb_0.1_O_2−δ_	Ce_0.9_Yb_0.1_O_2−δ_
a (Å)	5.4116(4)	5.4386(3)	5.4113(3)	5.4200(3)	5.4159(3)	5.3960(3)	5.4033(3)
Size (nm)	100(3)	31(2)	40(2)	76(2)	50(2)	57(4)	35(1)
+Co, a (Å)	5.4080(4)	5.4404(3)	5.4103(3)	5.4193(3)	5.4159(3)	5.3983(3)	5.4045(3)
Size (nm)	30(3)	16(1)	42(2)	44(1)	56(2)	61(1)	37(4)
Ionic radius (Å)	1.143 *	1.16	1.126	1.066	1.053	1.04	0.985

## Supplementary Materials

The following are available online at https://www.mdpi.com/2077-0375/10/7/148/s1: Figure S1: Rietveld refinement patterns of all the Ce_0.9_Ln_0.1_O_2−δ_ and Ce_0.9_Ln_0.1_O_2−δ_ +Co, with Ln= Eu, Gd, La, Pr, Tb, Yb. Figure S2: SEM image of CGO powder. Figure S3: Evolution of lattice volume of Ce_0.9_Pr_0.1_O_2−δ_ with temperature.
