# Supplementary material for: In Situ Raman Characterization of SOFC Materials in Operational Conditions: A Doped Ceria Study
Source: Membranes (Basel). 2020 Jul 10;10(7):148. doi: 10.3390/membranes10070148 (PMC7407173; doi:10.3390/membranes10070148)
Supplement: Supplementary file 1 [file membranes-10-00148-s001.pdf]

membranes

# Supplementary Materials: In Situ Raman Characterization of SOFC Materials in Operational Conditions: A Doped Ceria Study

**Cecilia Solís [1,2], María Balaguer [1] and José M. Serra [1,*]**

[1] Instituto de Tecnología Química (Universitat Politècnica de València–Consejo Superior de Investigaciones Científicas), Avenida de los Naranjos s/n. 46022 Valencia, Spain, Cecilia.Solis@frm2.tum.de (C.S.); mabara@itq.upv.es (M.B.)
[2] Heinz Maier-Leibnitz Zentrum (MLZ), TU München, Lichtenbergstr. 1, 85748, Garching, Germany,
* Correspondence: jmserra@itq.upv.es

Rietveld refinement patterns of all the $Ce_{0.9}Ln_{0.1}O_{2-y}$ samples as prepared (left column) and with Co addition (right column) and from top to the bottom $CeO_2$ and Ln= Eu, Gd, La, Pr, Tb, Yb.

Patterns show XRD patterns from 10 to 90°, observed data (red circles), calculated data (black line) and difference plot (blue line) together with the position of the Bragg reflections (green lines).

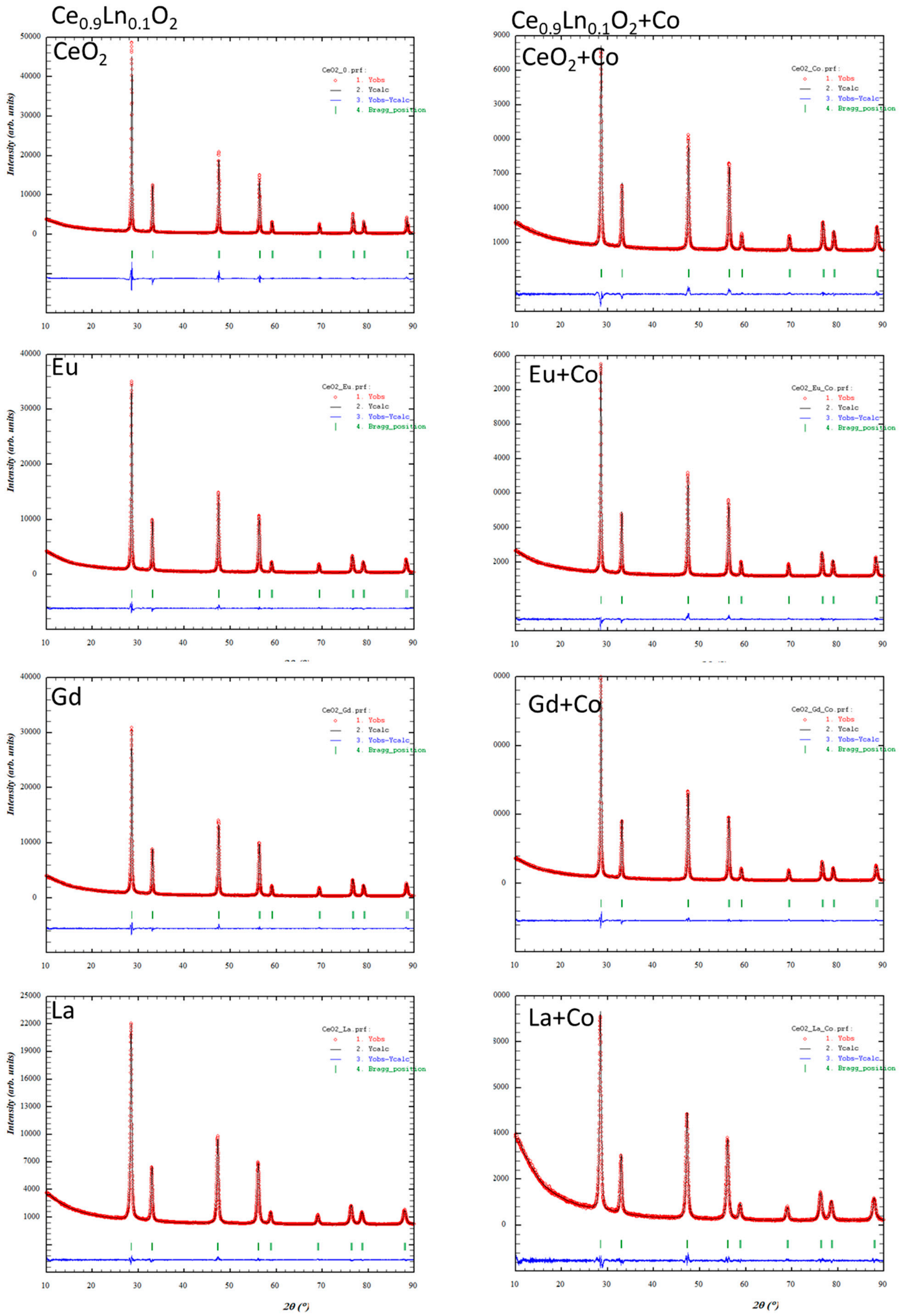

Ce0.9Ln0.1O2
Ce0.9Ln0.1O2+Co
CeO2
CeO2+Co
Eu
Eu+Co
Gd
Gd+Co
La
La+Co
CeO2_0.prf:
CeO2_Co.prf:
CeO2_Eu.prf:
CeO2_Eu_Co.prf:
CeO2_Gd.prf:
CeO2_Gd_Co.prf:
CeO2_La.prf:
CeO2_La_Co.prf:
1. Yobs
2. Ycalc
3. Yobs-Ycalc
4. Bragg_position
Intensity (arb. units)
2θ (°)

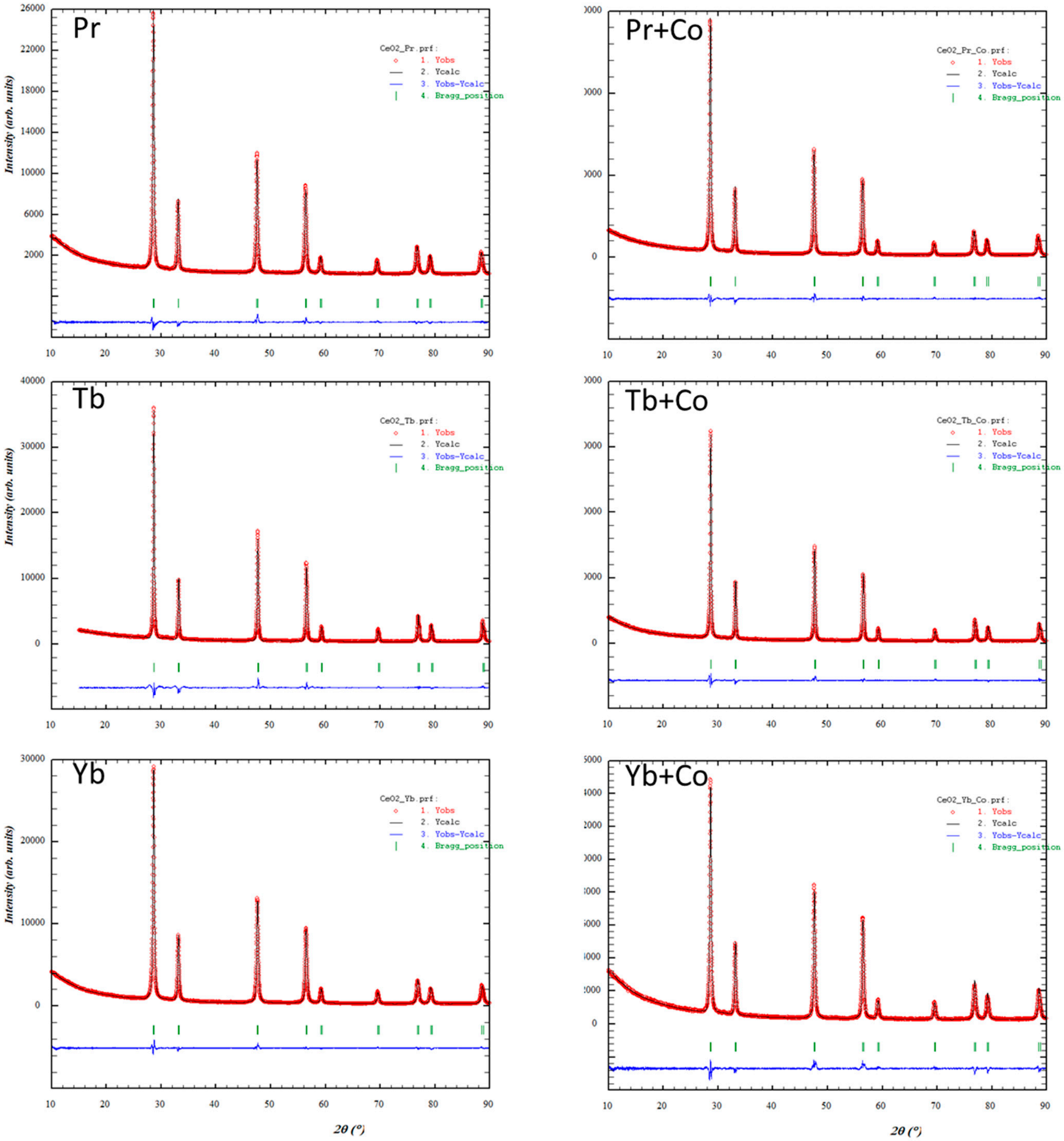


**Figure S1.** Rietveld refinement patterns of all the $Ce_{0.9}Ln_{0.1}O_{2-\delta}$ and $Ce_{0.9}Ln_{0.1}O_{2-\delta}$+Co, with Ln = Eu, Gd, La, Pr, Tb, Yb.

Scanning Electron Microscopy image of a CGO powder calcined at 600 °C in air, using a JEOL JSM6300 scanning electron microscope.

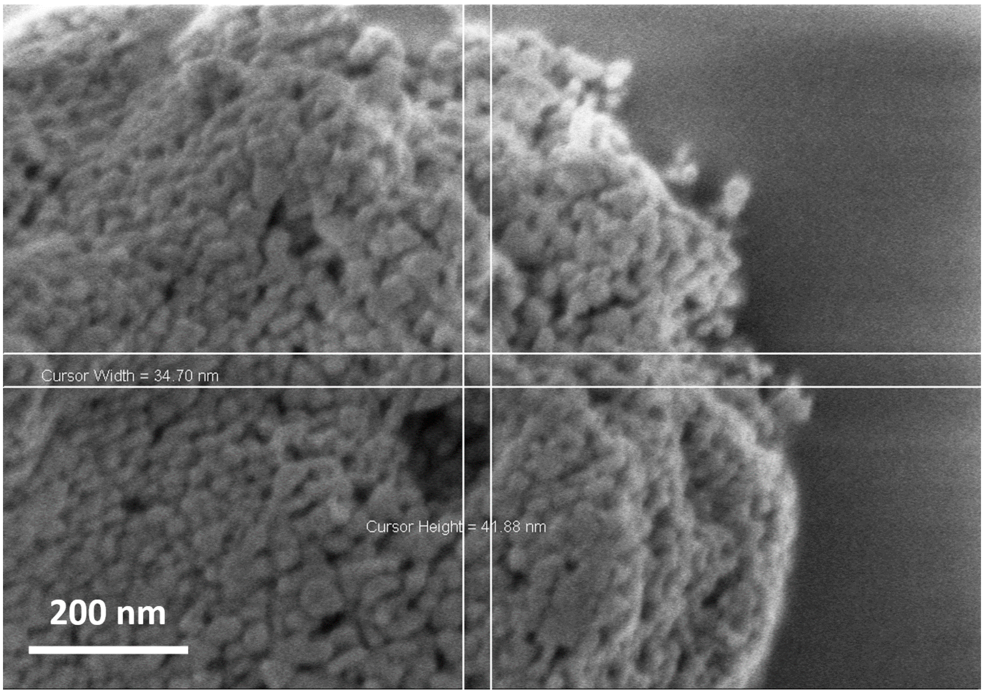


**Figure S2.** SEM image of CGO powder.

Evolution of lattice volume of $Ce_{0.9}Pr_{0.1}O_{2-\delta}$ calculated from lattice parameters extracted from high temperature XRD patterns in air. A change in the slope is due to the change on the oxidation state of the Pr with temperature.

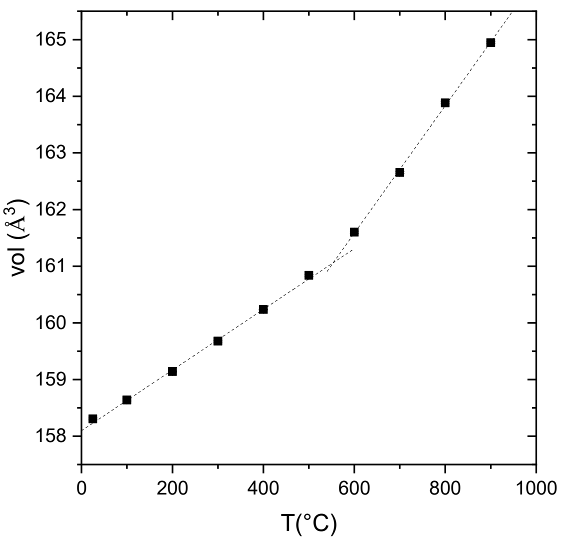


**Figure S3.** Evolution of lattice volume of $Ce_{0.9}Pr_{0.1}O_{2-\delta}$ with temperature.
